# Case of Paraplegia

**Published:** 1851-03

**Authors:** Wm. H. Gardner

**Affiliations:** Whitesboro, N. Y.


ART. II—*Case of Paraplegia.* Reported by WM. H. GARDNER, M. D., of Whitesboro, N. Y.

On the 21st Sept., 1847, I was called to R. P., a strong, healthy and intelligent boy, aged 13 years. I learned on inquiry that eight or ten days before,* he fell backwards from a lumber wagon, striking his back across a stick of wood, somewhere in the upper part of the lumbar region. He complained some at the time, but continued at work the remainder of that, and for four or five days following, without complaining, and as he said with little lameness. On the fifth or sixth day after the fall, when the weather was cold and damp, he was out in the field with other boys, amusing himself by throwing apples, and lay on the ground for some time, which was damp. During the evening, he was attacked with pain and lameness in his back, at the seat of the former injury, which had increased up to the time when I saw him. He was now, when still, suffering but little, but on the slightest motion of the body the pains became excruciating.

On examination, no perceptible marks or swelling were visible at the seat of the injury, and there was but little if any tenderness there. The constitutional symptoms were inflammatory. Vs. was practiced, together with cupping over the seat of the injury, and Tinct. of Colchicum, with cathartics, prescribed.

The next day, Sept. 22d, I saw him again. He expressed himself much better—had been able to get off the bed without assistance, and could move, or be moved, with much less suffering, but was complaining of a sensation of prickling in all that portion of his body below the seat of the injury.

Sept. 23d.—The sensation of prickling had increased, and there was partial loss of motion, the lower extremities not readily obeying the dictates of the will.

24th.—The symptoms of the previous day were aggravated, except the pain, which had gradually diminished. Cups were again applied, and as his bowels had not moved for the last day, a cathartic, or aperient, was administered, with directions to repeat the cathartic if necesary.

25th.—Dr. T. by my request saw him with me. His bowels had not responded to the medicine, nor had he passed his urine since the day before. In addition to this there was now total paralysis of all that portion

---

* In conversing with the parents and friends, I have taken the time that elapsed after the injury, before I saw him, from them. My own impression was that it was not so long.

of the body below the injury. The prickling sensation was gone, and he was free from pain. On examination, the bladder was felt fully distended, but he said he had not suffered at all from it. A catheter was now introduced without difficulty, and about three pints of urine brought away. He said he did not feel any sensation on its introduction.

A blister was now applied to the seat of the injury, and cathartics freely administered without the slightest effect, for the space of five or six days. During this time, injections with a pump syringe were resorted to, but after the first no fecal matter was brought away, and the liquid flowed back, as from a leather hose. A motion of the bowels at last followed the administration of a common Seidlitz powder, and copious stools, attended with flatus were discharged. After this, his bowels became regular, moving at proper and stated intervals without the aid of medicine; but yet he was not conscious of it at the time. During all this time, and for some days after, he voided no urine. It was regularly drawn twice a day, with the catheter. He never complained in the least of its introduction, declaring that he had no sensation about it. Sometimes when left a little more than twelve hours, he complained of an indefinite sense of uneasiness, which he could not locate, but which was at once relieved by the use of the instrument. About the tenth day from the paralysis and suppression, his urine commenced dribling away in small quantities, from one to two ounces at a time, as it was judged, and often repeated. The catheter was after this once or twice introduced, but the bladder was found empty. The quantity discharged at a time was gradually increased, and the interval between the discharges prolonged till it was nearly normal. But still both feces and urine were voided without his consciousness. From the commencement of the disease, not the slightest delirium had been manifested, his mind appearing ordinarily active. His appetite for food had not been craving, but yet he had daily called for, and taken some simple nourishment. In this respect he had gradually improved. Still the paralysis, below the injury, was perfect. On passing different substances down the course of the spine, sensation was perfect until it arrived to a certain point or line, where it ceased altogether, and this line seemed but a hair's breadth in width. On requesting him to try and move his feet, his uniform reply was "I can't try." Still, slight spasmodic twitchings were observable in the lower extremities. At the time of the injury he was fleshy, but had become somewhat emaciated, but *no disproportion was observable between the different portions of his body;* and the circulation was apparently as active in one part as the other.

About the first of October, a swelling was first observed upon the back, although it had been examined from time to time, situated upon both sides of the spinal column, but greater upon the left than the right. At this time the principal enlargement was between the lower portion of the left scapula and the adjoining vertebræ, but extended much above and below this point. This continued to increase in size, but more downwards than upwards, till it reached from the neck to the sacrum. Some two weeks after, it began to show symptoms of pointing, at or near the place of the original injury.

On the 16th of Oct., Dr. T. again visited the boy with me, and, on consultation, it was concluded to make an opening into the tumor, notwithstanding fluctuation could not be felt very distinctly. The point selected for the opening was on the left side against the upper part of the third lumbar vertebra, which happened to be immediately below the line of sensation. The skin was raised deliberately to make a valve, after which the knife was passed slowly inwards and upwards till matter was reached, at the depth of from one to one and a half inches. During all this time, the patient sat perfectly still, not even a muscle moved, nor was there a perceptible change in his breathing. He afterwards said, on being told that it was done, that he was not aware of it, and supposed that we were "only getting ready." The discharge was copious, and the pus healthy. After it had discharged, as was supposed, about a pint, the opening was closed with sticking plaster, and it was left till the next morning, when the plaster was removed, and the discharge permitted to go on. The swelling now disappeared, and the cavity soon filled up so that no marks of the abscess remained, except a small cicatrix at the external opening. The back now appeared natural; the spinous processes of the vertebræ could be distinctly traced both by the eye and the fingers, from one extremity to the other, and not the slightest curvature or displacement was discovered. But yet, in defiance of our predictions, neither motion or sensation had returned in the slightest degree. Still, when placed on a chair, he sat quite erect, using his hands to right himself, when by accident his body became bent in any direction. His appetite was now good, and he was allowed a generous quantity of nourishing food; he gained flesh rapidly, his countenance soon assumed the glow of health, and no perceptible difference could be seen between the growth of the different portions of his body. But the paralysis still remained. At this time he had a bed-sore near the promontory of the ischium, in which there was deep sloughing, and into which, from the position he assumed, foreign substances were often crowded so as to occasion bleeding and all the signs of irritation; still he never complained, and it healed rapidly. Other remedies were now resorted to successively, as friction, galvanism, blisters, strychnine, issues and setons, but all without any effect upon the paralysis. The blisters were so placed as to extend over the sensitive and insensitive portions, and healed as rapidly upon one as the other. The issues were made with caustic potash, immediately below the line of sensation, and he never uttered the least complaint. Both issues and setons healed readily. On irritating these sores when dressing them, and at other times, *the lower extremities were observed to move with a start*, such as is ordinarily witnessed on applying the hand suddenly to one in a healthy subject. These experiments were repeated, and the same effect was almost uniformly produced. Still he declared that he had no consciousness of any irritation about the sore, and that he did not know why his feet moved. Indeed, he was not conscious of the motion unless it was so great as to be transmitted to the upper portion of his body. On dashing cold water upon the lower extremities, the same motions were produced; and they in no way differed from those in ordinary cases. This was done at times when he did not see the water, and the same effect produced. On slapping the foot or leg smartly with the hand, like motions were produced and repeated for a great number of times. Similar motions were sometimes noticed without any apparent cause, but when the experiments were tried, they were uniform, or nearly so.

I continued to treat the patient till the 10th of April, 1848, visiting him occasionally. At this time he had acquired more than his usual amount of flesh, was in good spirits, and to a casual observer he presented the appearance of perfect health. Yet all my efforts to restore to him the use of his limbs, were totally unavailing, and he was still voiding his excrements without consciousness.

Soon after this, I learned that a respectable physician from an adjoining town had been called, who saw fit to prescribe for the patient without seeing or consulting me. He placed the boy, as I have been informed since, upon a bed, and enjoined perfect rest, in a recumbent position; but what else he did I do not know, as I never have had any conversation or correspondence with him upon the subject. In June, 1850, I was again called to treat another member of the family, when, as they were doing nothing, having lost faith in medicine, I took occasion to examine my former patient.

Dec. 30th, 1850, I again visited the boy, for the express purpose of examining him more closely, and to compare my own recollection of the case with that of his family and friends—which was found to agree. He has now grown to the stature of an ordinary sized man; and there is still little of any disproportion between the inferior and superior extremities. I thought the muscular portion of the legs was not quite as fully developed as the corresponding portion of the arms, but this was not as great as would be supposed to exist from the lack of exercise, and would not be noticed, without an examination and comparison. The legs are well shaped, and he has what would be called a good and healthy form. When I saw him before, I thought the legs were slightly flaccid to the feel, but now it was not perceived. Still the paralysis is as perfect as ever. He says that never, since the attack, has he experienced the least sensation in, or been able to move his lower extremities to the slightest extent, by his own volition—and he continues to pass his excrements without knowing it. On requesting him to try to move his foot, I was again met with the reply—"I can't try." On passing the back of a pocket knife blade down the course of the spine, I found the point at which sensation ceased was against the body of the second lumbar vertebra, and from thence extended around the body in the course of the nerves. I now repeated some of my former experiments. On dashing cold water on his legs, the same starts as above described were again witnessed, and repeated as often as the application was renewed. I then struck the limbs smartly with the flat of the hand, and repeated the blow for full twenty times, and every blow was followed with a like muscular contraction. This was tried on both limbs with the same effect. The person who had dressed the setons and issues informed me that the same phenomena was always witnessed on cleaning the sores and applying the dressings upon his back, till they were allowed to heal; and still, he never showed any signs of suffering from those that were located below the line.

The above case has been thus briefly detailed. Much doubtless is left out from lack of recollection, or for the reason it was not deemed of importance. But so far as it goes, I believe it perfectly accurate, having revived my memory by all the means within my reach, and compared it with that of others, with which it agreed.

The case may not be altogether devoid of interest in a medical or surgical point of view. The treatment can not now be very fully or accurately described, nor would it perhaps possess interest if it could. In restoring to the unfortunate boy the use of his limbs, it has been entirely unsuccessful. Slight differences of opinion might exist, in regard to the diagnosis and treatment of the case. But yet there can be little doubt that all, on an examination would agree, that nervous communication between the pain and lower portion of the body had been cut off or supended in some manner. If this be so, and we know of no reason to doubt it, the case demonstrates several physiological facts of some importance. It shows that the brain is not important to the proper performance of the functions of assimilation and disassimilation—or those of organic life. For in this case, for the space of more than three years, they have been carried on, as healthily below the second lumbar vertebra when the communication is stopped, as above it.

And it shows also that there *may be motion independent of the pain, or of any nervous communication with it.* Thus if the nerves are the only medium by which impressions are transmitted from one part of the body to another, there must be a set or system of nerves, not universally acknowledged, which preside over, and induce involuntary motions—a set that in their action, or functions, correspond to Marshall Hall's excito-motor or true spinal system. For in this case, motion—an apparent start, may at any time be produced by outward impressions, whose impulse is not transmitted to the brain, of which the mind takes no cognizance, and for the production of which no volition can be transmitted.

*Dec.* 31, 1850.

